# Temperature Dependence of Fracture Characteristics of Variously Heat-Treated Grades of Ultra-High-Strength Steel: Experimental and Modelling

**DOI:** 10.3390/ma14195875

**Published:** 2021-10-07

**Authors:** Jaroslav Pokluda, Ivo Dlouhý, Marta Kianicová, Jan Čupera, Jana Horníková, Pavel Šandera

**Affiliations:** 1 Faculty of Mechanical Engineering, Brno University of Technology, Technická 2, 616 69 Brno, Czech Republic; pokluda@fme.vutbr.cz (J.P.); idlouhy@ipm.cz (I.D.); cupera@fme.vutbr.cz (J.Č.); hornikova@fme.vutbr.cz (J.H.); 2 Faculty of Special Technology, Alexander Dubcek University of Trencin, Ku kyselke 469, 911 06 Trenčín, Slovakia; marta.kianicova@tnuni.sk; 3 Institute of Physics of Materials AS CR, Žižkova 22, 61662 Brno, Czech Republic

**Keywords:** ultra-high steel grades, tensile characteristics, fracture toughness, temperature dependence, modelling fracture

## Abstract

The temperature dependence of tensile characteristics and fracture toughness of the standardly heat-treated low-alloyed steel OCHN3MFA along with three additionally heat-treated grades was experimentally studied. In the temperature range of 〈−196; 22〉 °C, all the additional heat treatments transferred the standard steel from a high- to ultra-high strength levels even with improved tensile ductility characteristics. This could be explained by a reduction of the inclusion content, refinement of the martensitic blocks, ductile retained austenite content, and homogenization of the shape ratio of martensitic laths as revealed by metallographic, X-ray, and EBSD techniques. On the other hand, the values of the fracture toughness of all grades were found to be comparable in the whole temperature range as the cause of a high stress triaxiality in the pre-cracked Charpy V-notch samples. The values of the fracture toughness of the standard steel grade could be predicted well using the fracture model proposed by Pokluda et al. based on the tensile characteristics. Such a prediction failed in the case of additionally heat-treated grades due to the different temperature dependence of the fracture mechanisms occurring in the tensile and fracture-toughness tests. While the tensile samples fractured in a ductile-dimple mode at all temperatures, the fracture-toughness specimens exhibited a transition from the ductile to quasi-brittle fracture mode with decreasing temperature. This transition could be interpreted in terms of a transfer from the model proposed by Rice and Johnson to the model of Tvergaard and Hutchinson.

## 1. Introduction

The global engineering approach to the assessment of the integrity of flawed mechanical structures directly results from linear or elastic-plastic fracture mechanics describing the fracture resistance in terms of the fracture toughness (FT) represented by the critical stress intensity factor *K*_Ic_ or the critical J-integral *J*_Ic_ [1,2]. The transfer of the data from laboratory specimens to engineering structures and components is not necessarily straightforward due to the constraint effects and, therefore, the two-parameter fracture mechanics is more relevant [3]. Nevertheless, the values of *K*_Ic_ or *J*_Ic_ are, besides the values of the Charpy-notch toughness (impact energy), crucial characteristics enabling a mutual comparison of engineering materials with respect to their fracture resistance. The FT tests remain the only relevant methods for determination of FT, but they are rather complicated and expensive. It is thus worthwhile to investigate the possibility of theoretical predictions of FT values using the data from much simpler and cheaper tests. Such predictions are usually based on models dealing with local approaches to fracture, both deterministic and probabilistic, which rely on the fact that it is possible to model macroscopic fracture behavior in terms of numerically calculated elastic-plastic stress-strain in the process zone combined with local fracture criteria.

Many advanced ceramics and composites as well as bcc metallic materials at lower temperatures exhibit brittle (cleavage) fracture. An important group of local cleavage-fracture models were proposed by the pioneering work of Beremin [4] and assume that fracture is initiated when a microcrack ahead of a crack front is subjected to a critical value of the Weibull stress and relies on a numerically calculated evolution of this local stress with the macroscopic applied stress (e.g., [5,6]). Kotrechko et al. [7] focused on the mechanisms of crack nucleation in ferritic steels by incorporating both the microscopic stress induced by elastic deformations and the stress caused by dislocation pile-ups into the probabilistic model. These statistical approaches predict the probability of brittle fracture for the given applied *K*-value along with the upper and lower bounds of the temperature dependence of FT. However, their application is rather cumbersome since it demands the determination of many material parameters from microstructure samples and numerous FT and tensile tests at multiple temperatures. Therefore, Yankova et al. [8] recently proposed a thinning function for the temperature dependence of cleavage initiators that promises a reduction of requested FT tests. Recently, the strain-gradient fracture mechanics coupled with atomistic approaches was utilized to eliminate the stress singularity at the crack tip and to predict FT for components sized in the entire range from macro to nano (e.g., [9]). This method is numerically complex and time consuming and, up to now, its verification was done only for single crystals of several pure metals and ceramics [10].

In high-strength steels with a basic martensitic structure and alloys with bcc matrix strengthened by fine particle dispersion, the specific transition behavior can be observed. In the upper part of the transition, plastic crack-tip blunting occurs before the unstable fracture associated with the microvoid coalescence mechanism and a ductile-dimple morphology of the fracture surfaces. With decreasing temperatures, fraction of the cleavage facets on the fracture surface dispersed between ductile-dimple areas increases and the microvoid coalescence starts to be controlled by decohesion of the particle/matrix interfaces and, due to a very low interparticle spacing, the stage of void growth is suppressed. However, shallow ductile dimples often remain observable even at very low temperatures. This was also the case of the high-strength steel OCHN3MFA (Russian GOST nomenclature) investigated in this work and, consequently, the local ductile-fracture models seemed to be more relevant for a description of its fracture process. Such models can be based either on the plastic work consumed in the plastic zone till the onset of fracture or on a separation energy related to the rupture of ligaments between the voids as reported in the classical works of Peel and Forsyth [11], Hahn and Rosenfield [12,13], McClintock [14], and Gurson [15]. Based on these assumptions, ductile fracture models were developed by Rice and Johnson [16], Pokluda et al. [17,18,19], and Tvergaard and Hutchinson [20]; see the Appendix A for more detail. These models are considered in this article since they enable a straightforward prediction of FT values using rather simple available tensile and microstructural material data. Some more recent works and reviews on ductile fracture [21,22,23], useful for comparative reasons and knowledge extension, are also worthwhile to mention here.

The fracture-toughness samples of the originally (standardly) heat-treated steel OCHN3MFA exhibited a ductile fracture morphology when tested at room temperature [24]. Some preliminary results indicated that, for this steel grade, the model of Pokluda and Šandera [18,19] could reasonably predict the *K*_Ic_ (or *K*_Jc_) values even at lower testing temperatures [25]. Industrial interest was then expressed for further verification of such a conclusion along with mechanical testing of additionally heat-treated steel grades. Therefore, the experimental research presented in this article was focused on the temperature dependence of the basic mechanical properties, FT, and temperature dependence of FT and the related fracture mechanisms as several OCHN3MFA steel grades. The capability of selected fracture models to predict the temperature dependence of FT and the related fracture mechanisms is also reported.

## 2. Material, Heat Treatment, and Microstructure

The high-strength low-alloy steel OCHN3MFA of the original (commonly applied) heat treatment consisting of quenching and stepwise tempering, along with another three grades of additional quenching and tempering at different temperatures were investigated. The chemical composition of the steel is displayed in Table 1. The heat treatment of original grade-conventional additionally heat-treated grades is shown in Table 2. The grades exhibited the following average values of Vickers hardness (5 indentations for each treatment): original treatment–502 HV, tempering 160 °C–725 HV, temp. 200 °C–706 HV, temp. 250 °C–696 HV.

**Figure 1:**
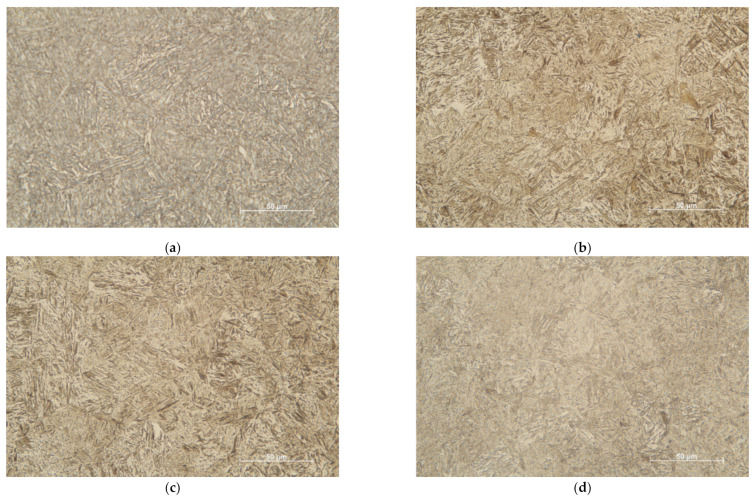
Microstructures of the steel grades used in this study—(**a**) the original treatment, (**b**) the additional treatment (tempering at 160 °C), (**c**) the additional treatment (200 °C), and (**d**) the additional treatment (250 °C).

**Figure 2:**
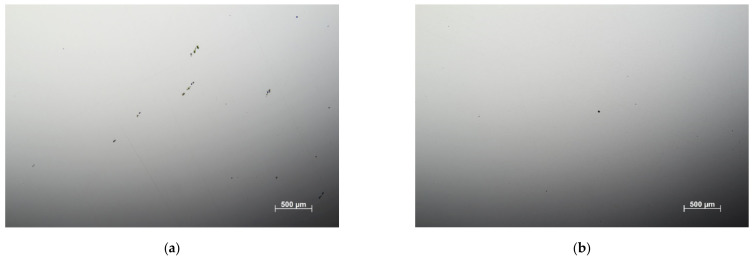

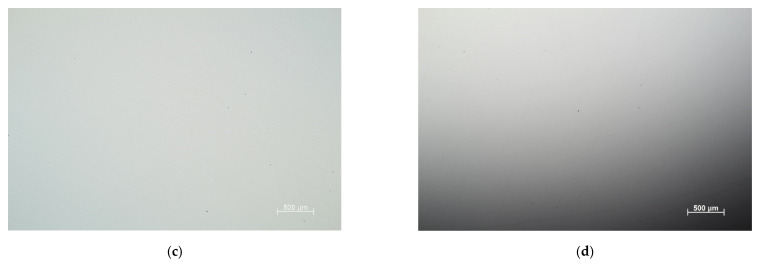
Representative examples of the inclusion content in the OCHN3MFA steel: (**a**) original heat treatment, (**b**) additional treatment (160 °C), (**c**) additional treatment (200 °C), and (**d**) additional treatment (250 °C).

The microstructures related to individual steel grades are documented in Figure 1 as obtained by a classical metallography technique. The microstructure of all grades consisted of fine tempered martensite laths containing low or no tetragonality martensite and carbide particles. It is well known that the population of carbides (not visible in Figure 1) depends on the tempering temperature. At the low tempering temperatures corresponding to the additionally treated grades, the martensitic matrix contains Fe_2,7_C carbides, which change to the cementite Fe_3_C particles at higher tempering temperatures applied to the originally treated samples (e.g., [26,27]). All originally and additionally heat-treated samples were machined from the same (single) forged structural component. Moreover, observable changes in the number and the size of inclusions in steels usually start only at annealing temperatures higher than 1250 K (e.g., [28]), which were not reached during the additional heat treatment. However, recent observations ([29]) indicated that repeated annealing, quenching, and tempering can significantly reduce both the size and the number of inclusions. The content of impurities in all grades is shown in Figure 2. One can see that the additional heat treatment really caused a reduction of the number of inclusions, thus confirming the conclusion of the paper [29].

The volume fraction of the retained austenite (RA) in steel grades was measured by the X-ray diffractometer system EMPYREA and the measurement program PANalytical with the following result: the additional treatment (160 °C) contained 11.2% RA; the additional treatment (200 °C) 9.4% RA, the additional treatment (250 °C) 3.2% RA, and the original treatment 0% RA. As expected, the percentage of RA decreased with the increasing final tempering temperature. With respect to [30], the retained austenite is expected to be thermo-mechanically stable in the elastic loading range and service temperatures between 0 and 250 °C.

**Figure 3:**
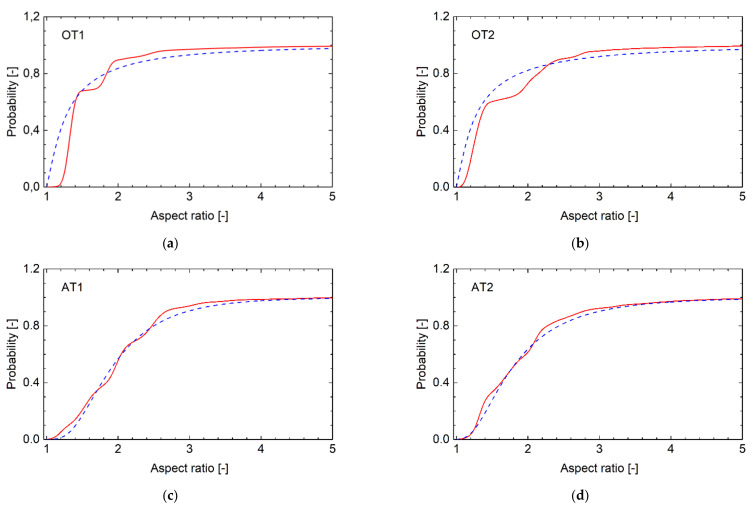
The experimental cumulative distribution function (CDF_ex_, full line) of the lath aspect ratio along with the log-normal approximation (CDF_LN_, dashed line): (**a**,**b**) original samples OT1 and OT2; (**c**,**d**) additionally treated samples AT1 and AT2.

To find out possible differences in the structure of martensitic laths, the electron backscatter diffraction (EBSD) was employed on two originally treated (OT1, OT2) and two additionally treated (AT1:160 °C, AT2:250 °C) samples using FEG-SEM Carl Zeiss ULTRA PLUS (Carl Zeiss NTS GmbH, Oberkochen, Germany) equipped with an EBSD detector HKL Nordlys (Oxford Instruments, High Wycombe, UK). The EBSD data were collected by AZtech software and processed by Channel 5 software (both Oxford Instruments). A quantitative analysis of the shape and crystallographic orientation of nearly 3000 martensitic laths, digitally identified within the area defined by the matrix of 768 × 1024 pixels, was performed in each sample. The laths were approximated by the best-fit ellipses characterized by their area and aspect ratio (main/minor axes). All values of the aspect ratio were inside the range of (1; 15), which was divided into 100 equal segments. Inside each segment, the number of aspect ratios was weighted proportionally to the area of the related laths. Such obtained cumulative experimental dependences were normalized to obtain the cumulative distribution function (CDF_ex_), with values lying in the range of 〈0, 1〉, plotted for all samples in Figure 3. Observe that the CDF_ex_ functions of the AT samples are smoother than those of the OT ones, exhibiting more wavy shapes. The values of the weighted arithmetic mean *A*_ex_ and the median *M*_ex_ obtained from CDF_ex_ functions are shown in Table 3, showing that both the *A*_ex_ and *M*_ex_ values for the OT and AT samples are significantly different. The CDF_ex_ functions were fitted by the three-parameter log-normal distribution *F*(*X*):

$$\frac{dF\left(X\right)}{d\left(X\right)}=f\left(X\right)=\frac{1}{\left(X-\lambda \right)\sigma \sqrt{2\pi }}\;\exp\frac{{\left[\ln\left(X-\lambda \right)-\mu \right]}^{2}}{2\sigma^{2}}$$

where *f*(*X*) is the probability density function. When selecting *λ* = 1 (the minimum of the aspect ratio), the parameters *μ* and *σ* were determined (see Table 3) and the related CDF_LN_ functions plotted in Figure 3 to see their high consistency with the experimental CDF_ex_ functions. A difference between the values of both parameters *μ* and *σ* for the OT and AT samples is also clearly visible in Table 3. In general, the analysis showed that the aspect-ratio values in the AT samples were rather uniformly distributed inside the range of 〈1, 15〉, while high discontinuities of the package shapes existed in the microstructure of the OT specimens.

**Figure 4:**
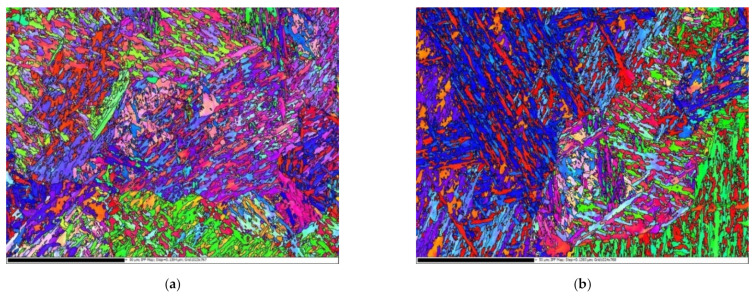

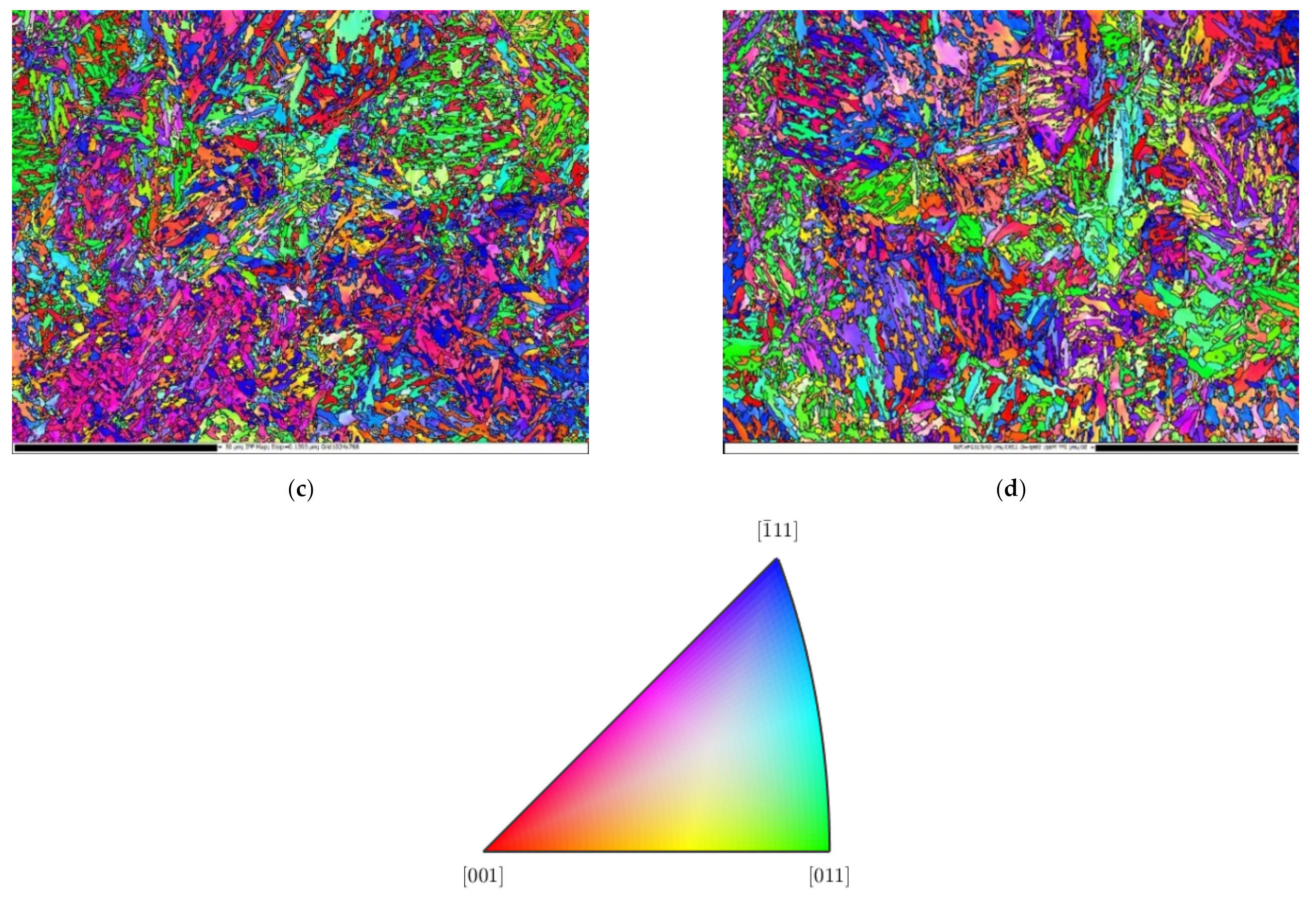
The EBSD crystallography maps: (**a**,**b**) the original heat treatment; (**c**,**d**) additional heat treatments (160 and 250 °C).

The maps of the crystallographic orientation of the laths depicted in Figure 4 show that the OT microstructure consisted of extended areas (martensitic blocks or even packets) with preferable [111] + [001] (blue + red) and [110] + [001] (green + red) orientations while the orientational distribution of the AT laths was more spatially refined and homogeneous, i.e., within smaller blocks. This means that, in the OT grades, more extended channels and corridors with a rather uniform crystallographic orientation were available for easy movement of dislocations.

## 3. Tensile Characteristics of Steel Grades at Various Temperatures

**Figure 5:**
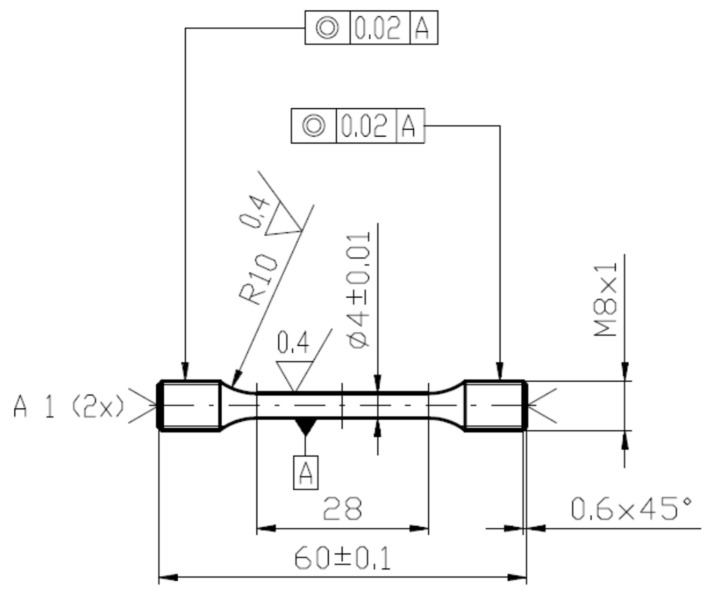
The scheme of tensile specimens.

Tensile tests were performed using the universal testing machine Zwick/Roell Z250 equipped with a cryogenic chamber. The testing temperatures were 22, 0, −20, −60, −80, −100, −120, and −196 °C, and the cross-head rate was 2 mm/min. The temperature was controlled by a thermocouple near the head of the tensile samples (bars), the scheme of which is shown in Figure 5. The samples were tempered at the requested temperature with an accuracy of ±1.5 °C for 20 min. The elongation was measured by the extensometer Multisens synchronized with the movement of the loading frame and the commercial software TestXpert was employed for a determination of basic tensile characteristics. The dimensions of fractured bars as the ultimate elongation and the smallest diameter of the neck were measured using the microscope Mitutoyo.

**Figure 6:**
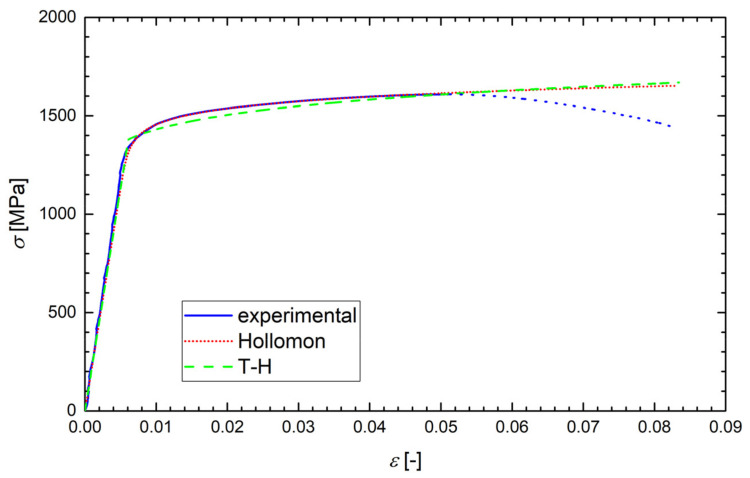
The experimental diagram of the true stress *σ* vs. true strain *ε* with its Hollomon and T-H approximations for a sample OT05 of the original grade tested at −60 °C. The dotted line corresponds to the invalid range of data.

**Figure 7:**
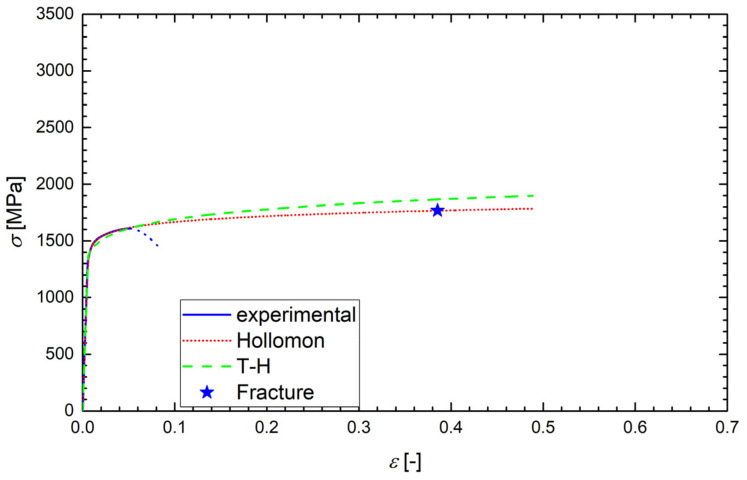
The experimental tensile diagram of the true stress *σ* vs. true strain *ε* for the sample OT05 (from Figure 6) with its Hollomon and T-H approximations plotted up to the fracture strain indicated by the star. The dotted line corresponds to the invalid range of data.

**Figure 8:**
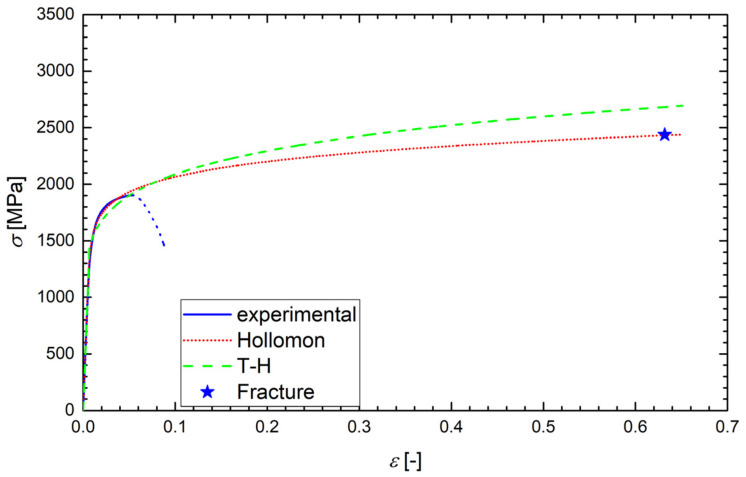
The experimental tensile diagram of the true stress *σ* vs. true strain *ε* for the sample AT(250)5 of the additionally treated grade tested at 22 °C. The Hollomon and T-H approximations are plotted up to the fracture strain indicated by the star. The dotted line corresponds to the invalid range of data.

**Figure 9:**
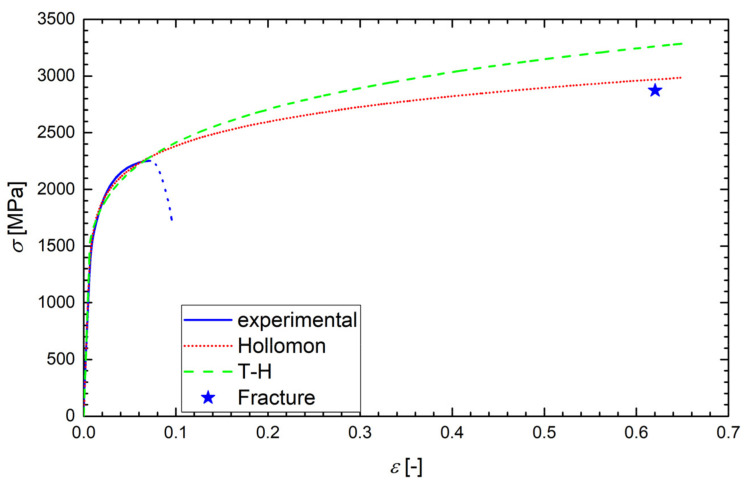
The experimental tensile diagram of the true stress *σ* vs. true strain *ε* for the sample AT(200)2 of the additionally treated grade tested at −120 °C. The Hollomon and T-H approximations are plotted up to the fracture strain indicated by the star. The dotted line corresponds to the invalid range of data.

According to the ASTM E6-03 standard, the true stress *σ* was calculated as the instantaneous normal stress, based on the instantaneous cross-sectional area, *A*. Since the strain data from the tensile test were obtained just using the extensometer, the values of *A* were not directly measured. Up to the onset of necking, however, the true stress could be calculated as *σ* = *F*/*A*_0_ (1 + *ε*), where *F* is the load and *A*_0_ is the original cross-section. Such a formula was also used after the onset of necking (after reaching the ultimate stress), but in that range, it did not give relevant true stress values and, obviously, the true strain data (*ε* = ln(1 + *ε*_eng_), where *ε*_eng_ is the engineering strain) were not correctly calculated. Although this range of data was never used in the calculations, it is still plotted in Figure 6, Figure 7, Figure 8 and Figure 9 and marked by dotted lines as invalid data. However, we could determine the true stress and true strain (*σ*_f_, *ε*_f_) at the point related to the final fracture of the specimen by measuring the diameter *d* of the minimum cross-section area: *σ*_f_ = *σ*_eng_⋅(*d*_0_/*d*)^2^ and *ε*_f_ = 2ln(*d*_0_/*d*), where *d*_0_ is the original specimen diameter. Such calculated values of *σ*_f_ and *ε*_f_ correspond to the stars plotted in Figure 7, Figure 8 and Figure 9.

Examples of the measured tensile true stress–strain curves for selected grades and testing temperatures are displayed in Figure 6, Figure 7, Figure 8 and Figure 9. The tensile samples of the original grade (the current construction material) were denoted as OTx, where x = 1, 2, …, is the number of a sample. The tensile specimens of grades additionally treated by quenching and tempering at 160, 200, and 250 °C were denoted as AT(160)x, AT(200)x, and AT(250)x, respectively. The basic mechanical properties of the Young modulus *E*, yield strength *σ*_y_, (0.2% proof stress), ultimate strength *σ*_u_, uniform elongation *A*_u_, elongation to fracture *A*_f_, reduction in area *RA*, and fracture strain *ε*_f_ are displayed for all steel grades and testing temperatures in Table 4, along with the values of *d*_0_ and *d*. One can see that the originally heat-treated OCHN3MFA steel can be assigned to the category of high-strength steels, with *σ*_u_ in the range of 1300–1800 MPa [31]. On the other hand, all the additionally heat-treated grades fall into the range of ultrahigh-strength steels, with *σ*_u_ higher than 1900 MPa [32]. This strength improvement can be related to the refinement of martensitic blocks with a nearly uniform crystallographic orientation as identified by the EBSD measurement. In terms of the Hall–Petch concept, this means shorter segments, mean free paths, and pile-ups of dislocations and, consequently, a higher yield stress. A further reason was the reduction of both the number and the size of inclusions.

It should be emphasized that the additional heat treatment substantially improved not only the strength level but also dramatically raised the ductile characteristics *RA* and *ε*_f_ of the material at all testing temperatures. At the lowest testing temperature of −196 °C, indeed, the values of the ductile characteristics of all AT specimens became even three times higher than those of the OT specimens. This was due to the two-phase microstructure of the AT grades, particularly in the ductile phase of retained austenite (absent in the OT grades). Moreover, higher discontinuities identified in the lath shapes of the OT microstructure could produce some strain incompatibility during the tensile deformation, thus reducing the fracture strain of the OT samples compared to that of the AT specimens. A third reason could be seen in the reduction of the inclusion content.

In general, the tensile characteristics of the additionally treated grades were comparable to high-end materials in the category of low-alloyed steels, such as AISI 4340 or 300 M, although the production technology of the latter steels includes, unlike that of the OCHN3MFA steel, purifying technologies, such as electro-slag refining or vacuum arc remelting [33,34]. Obviously, these technologies were, at least partially, substituted by the additional heat treatment.

The application of local approaches to the prediction of FT demands identification of the appropriate approximations of the true stress vs. true strain curve *σ-ε* by the Hollomon function *σ* = *Aε*_p_^n^ (*ε*_p_ is the true plastic strain) and by the Tvergaard and Hutchinson approximation in the form of Equation (A9b) in the Appendix A, hereafter called T-H approximation. The fitting parameters *A*, *E*, *n*, and *N* were determined in the following way.

### 3.1. Approximation in the Elastic Region

The yield stress *σ*_y_ corresponds to the plastic strain *ε*_p,y_ = 0.002 and the Young modulus can be obtained using two values *σ*_y_ and ε_y,e_ = ε(*σ*_y_) − 0.002 as *E* = *σ*_y_/*ε*_y,e_. In the whole stress range 0 ≤ *σ* ≤ *σ*_max_ then, the elastic part of strain corresponds to *ε*_el_ = *σ*/*E* and the plastic part to *ε*_p_ = *ε* − *ε*_el_.

### 3.2. Hollomon Approximation

For stress values *σ* ≥ *σ*_y_ and the plastic part of strain, the experimental dependence *σ* vs. ε was approximated by the function *σ* = *A ε*_p_^n^. The experimental data in the range *σ*_max_ ≥ *σ* ≥ *σ*_y_ were then fitted by the linear function log(*σ*) = log(*A*) + *n* log(*ε*_p_) using the least square method to obtain the values of *A* and *n*.

### 3.3. T-H Approximation

The Young modulus in the elastic range *σ* ≤ *σ*_y_ was again obtained as *E* = *σ*_y_/*ε*_y,e_. The approximation $\varepsilon =\frac{\sigma_{y}}{E}{\left(\frac{\sigma }{\sigma_{y}}\right)}^{\frac{1}{N}}$ in the elastic-plastic range *σ*_max_ ≥ *σ* ≥ *σ*_y_ (see Equation (A9) in the Appendix A) can then be written as a one-parametric function $\frac{\varepsilon }{\varepsilon_{y,e}}={\left(\frac{\sigma }{\sigma_{y}}\right)}^{\frac{1}{N}}$. The experimental data were then fitted by the direct proportionality $N\log\left(\frac{\varepsilon }{\varepsilon_{y,e}}\right)=\log\left(\frac{\sigma }{\sigma_{y}}\right)$ using the least square method to obtain the value of *N*.

Such determined values of *A*, *n*, and *N* for all grades and testing temperatures are summarized in Table 5. Examples of the regression curves for all grades and various testing temperatures are drawn in Figure 6, Figure 7, Figure 8 and Figure 9. One can see a good matching of the experimental and approximated Hollomon and T-H curves up to the maximum uniform strain *ε*_max_ = 0.05 corresponding to the maximal loading force (ultimate strength) as shown in Figure 6. For higher strain values, the experimental dependence obtained by an extensometer naturally does not match the strain localization during the necking process. However, both approximations reproduce the localized strain well even up to the specimen fracture, which can be seen from the position of the point (star) corresponding to the fracture stress *σ*_f_ and fracture strain *ε*_f_ as obtained from the neck geometry of the fractured specimens in Figure 7, Figure 8 and Figure 9. Indeed, these points lie close to both approximation curves, particularly to the Hollomon curve.

## 4. Fracture Toughness of Steel Grades at Various Temperatures

**Figure 10:**
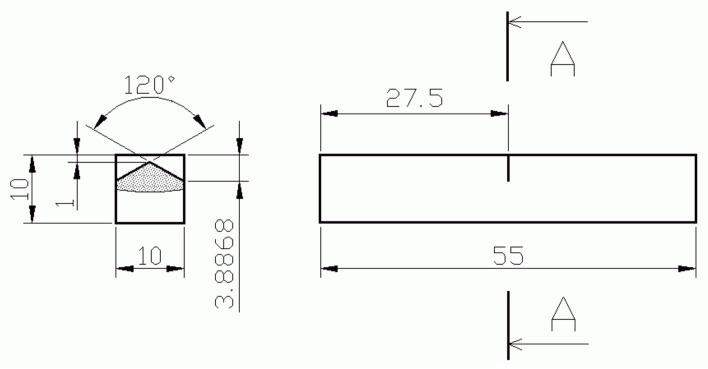
The specimen geometry used for the fracture toughness test.

The FT tests and their evaluation was performed according to the ISO standard [35]. The pre-crack generation was performed using the machine Zwick/Roell Z50 in four-point bending at room temperature. The chevron notch was cut by electric discharge machining. The FT of samples was then tested in three point bending with a 40 mm span of rollers. To reach the corresponding test temperature, the tests were carried out by applying a crosshead speed 1 mm/min in the cryogenic chamber of the Zwick/Roell Z50 (cooling by the liquid nitrogen vapors). In the load–displacement dependence, either the maximum load *F*_C_ (brittle fracture) or the load *F*_5_ (small preceding plasticity) were identified. The latter value was specified by the intersection of the linear-elastic part with the secant inclined by 5% tan α. After identification of the initial crack length *a*_0_ by means of the image analyses from optical macrographs of the fracture surfaces, the preliminary value *K*_Q_ of FT was calculated using the values *F*_C_ or *F*_5_. If *K*_Q_ fulfils the conditions of small-scale yielding and plane strain, then *K*_Q_ = *K*_Ic_ as the valid linear-elastic FT. If these conditions were not fulfilled but the condition of a small subcritical crack advance Δ*a* (image correlation) was proved, the *K*_Jc_-value was determined as:

$$K_{Ic}=\sqrt{\frac{E\cdot J_{Ic}}{1-\nu^{2}}},$$

where *J*_*Ic*_ is the critical value of the *J*-integral and ν is the Poisson ratio. If even the condition of a small subcritical crack advance failed, the calculated value was only considered a rough FT value and denoted as *K*_Ju_. The geometry and dimensions of FT samples are shown in Figure 10. The specimens of the original material grade are denoted FT(O)x, x = 1, 2,…, and the samples of additionally heat-treated grades, quenched and tempered to 160, 200, and 250 °C, are denoted as FT(160)x, FT(200)x, and FT(250)x, respectively.

**Figure 11:**
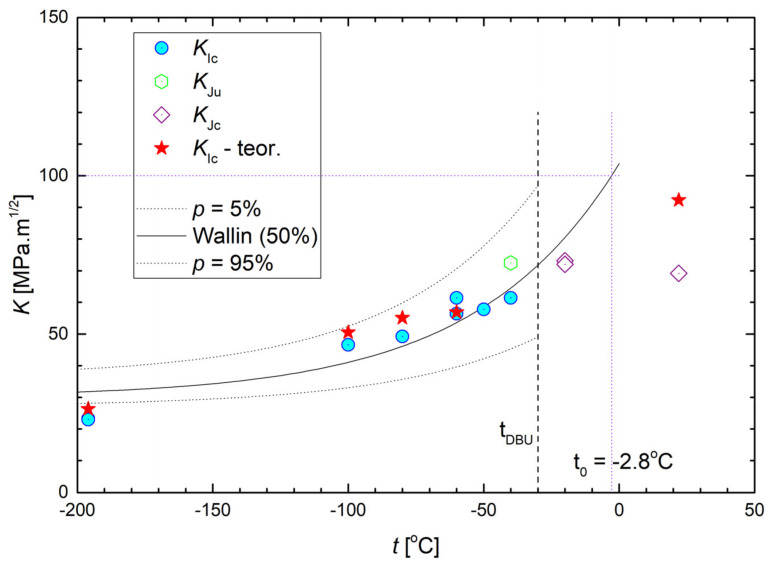
Temperature dependence of the measured and predicted fracture toughness of originally treated F(O) samples.

The values of FT for the original FT(O) specimens at all testing temperatures are collected in Table 6 and plotted in Figure 11. All the *K*_Ic_ values lying below the brittle-ductile transition temperature t_DBU_ = −30 °C are valid values of the linear-elastic plane-strain (LEPS) FT, except for the value denoted *K*_IJ_ at −40 °C that was recalculated from the critical J-integral value. All the values lying above the t_DBU_ temperature and denoted *K*_Ju_ are invalid. The temperature dependence of valid *K*_Ic_ data, corrected for the size effect, follows the concept of the Master curve well according to ASTM E1921-97 [36]. The Master curve is described by the formula:

$$20+\left\{11+77\exp\left[0.019\left(T-T_{0}\right)\right]\right\}{\left[\ln\left(\frac{1}{1-p}\right)\right]}^{\frac{1}{4}},$$

where *T*_0_ is the reference temperature and *p* is the cumulative probability of fracture. In our case, *T*_0_ = −2.8 °C, *p* = 0.05, and *p* = 0.95 stands for the lower and the upper bound curve, respectively, and *p* = 0.5 for the median curve.

On the other hand, all the data in the temperature range 〈−196; 22〉 °C displayed for the additionally treated grades in Table 6 are valid values of the linear-elastic FT. Unlike the tensile characteristics, the FT values of all grades were generally quite similar when compared at a given testing temperature. This was a consequence of a highly triaxial stress state at the crack front as discussed in the next section in more detail.

## 5. Morphology of Fracture Surfaces

**Figure 12:**
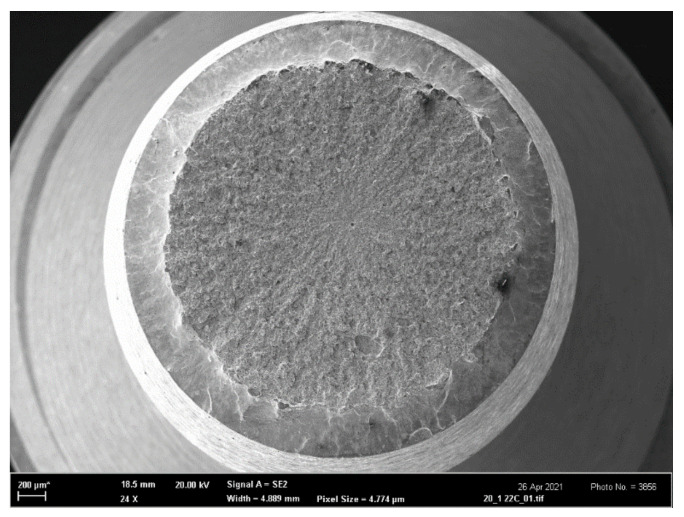
Fracture surface of the additionally treated tensile sample AT (200) tested at 22 °C. The reduction of the specimen thickness due to necking and the slant circular shear ring surrounding the flat central fracture area are clearly visible.

**Figure 13:**
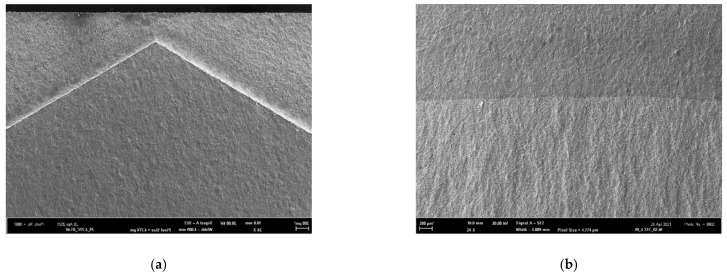
Examples of the macro-morphology of the fracture surfaces of samples FT (X): (**a**) the fatigue crack growth area inside the Chevron notch (middle and bottom part); (**b**) the boundary between the fatigue crack growth area (upper part) and the fast fracture area (bottom part).

The fracture morphology of all samples was studied in the scanning electron microscope (SEM) Zeiss-FEG SEM ULTRA PLUS. Macroscopic SEM pictures of typical fracture surfaces of the tensile and FT samples are depicted in Figure 12 and Figure 13, respectively. The thickness reduction by necking of the tensile specimen and the circular shear ring surrounding the flat central fracture area are clearly visible in Figure 12. The morphology of the fatigue crack starting inside the chevron notch is depicted in Figure 13a, while the boundary (fatigue crack front) between the fatigue crack growth area (upper part) and the fast fracture area (bottom part) is clearly visible in Figure 13b.

**Figure 14:**
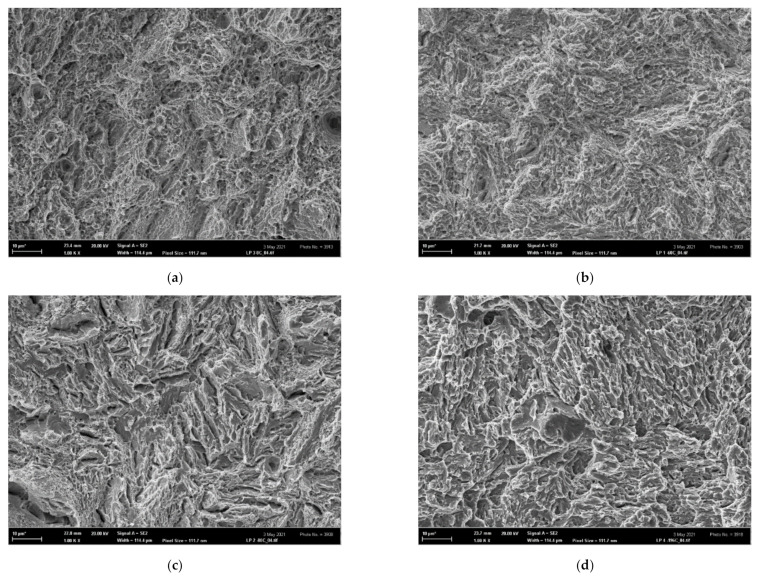
The morphology of tensile OT samples: (**a**) ductile dimple, tested at 22 °C, (**b**) ductile dimple, tested at −60 °C, (**c**) mixed ductile-brittle, tested at −80 °C, (**d**) quasi-brittle, tested at −196 °C.

**Figure 15:**
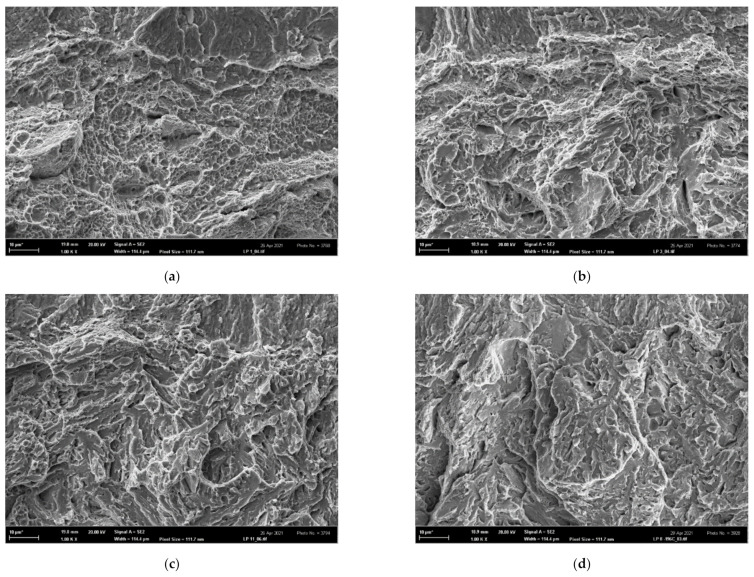
The morphology of fracture-toughness samples FT(O)x; (**a**) FT(O)12 tested at 22 °C, ductile dimple morphology, (**b**) FT(O)6 tested at −60 °C, ductile dimple morphology near the fatigue/final fracture boundary, (**c**) FT(O)4 tested at −80 °C, mixed ductile-brittle morphology near the fatigue/final fracture boundary, and (**d**) FT(O)1 tested at −196 °C, quasi-brittle morphology.

The microscopical fracture surfaces of all tensile OT specimens in the temperature range 〈−60; 22〉 °C were of a typical ductile dimple morphology as shown in Figure 14a,b. The fracture surfaces at −80 °C in Figure 14c exhibited features of microplasticity combined with some small cleavage facets, thus creating a mixed ductile/brittle morphology. At the testing temperature of −196 °C, a higher number of cleavage facets already occurred, and the morphology obtained a more brittle character (Figure 14d). As shown in Figure 15a,b, the morphology of FT(O) specimens in the temperature range 〈−60; 22〉 °C was ductile dimple in correspondence with that of the OT samples. Note that the ductile-dimple area adjacent to the fatigue/final fracture boundary is the relevant morphology for the FT(O) specimen tested at −60 °C (Figure 15b) since it corresponds to the crack-tip process zone. In the FT(O) specimen tested at −80 °C, the mixed ductile-brittle morphology appeared near the fatigue-final fracture boundary as depicted in Figure 15c. At the lowest temperature of −196 °C, the fracture surface of the FT(O) sample again exhibited a more brittle character in correspondence with that of the tensile OT sample, as shown in Figure 14d and Figure 15d.

In general, there was good correspondence between the fracture morphology of the OT and FT(O) specimens at all testing temperatures, which documented a similarity in the fracture mechanisms operating in the tensile and FT tests in the whole temperature range.

**Figure 16:**
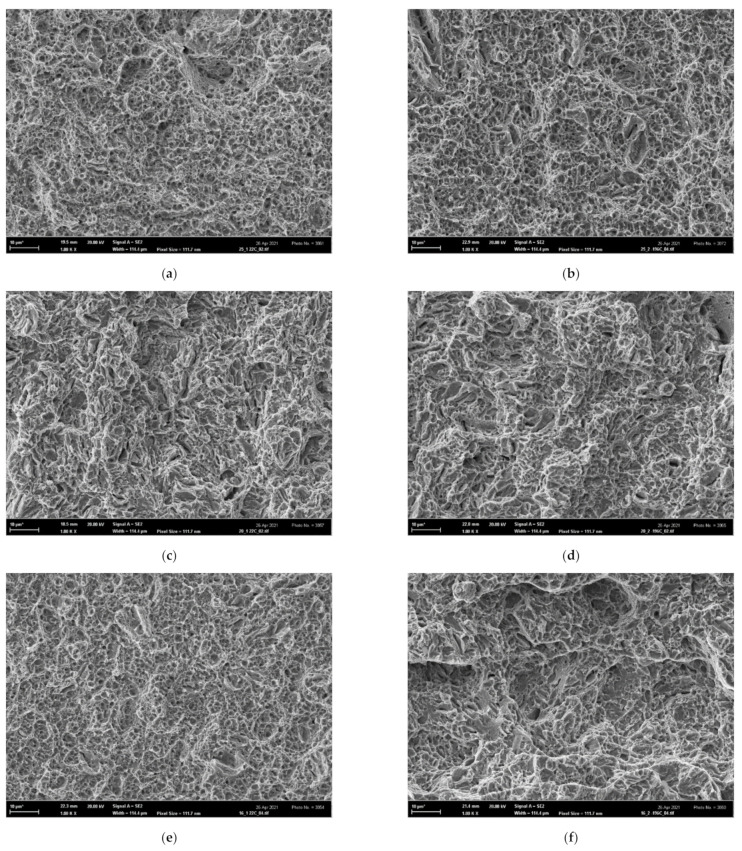
Ductile dimple morphology of tensile AT(X)x specimens: (**a**) AT(250)5 tested at 22 °C, (**b**) AT(250)1 tested at −196 °C, (**c**) AT(200)5 tested at 22 °C, (**d**) AT(200)1 tested at −196 °C, (**e**) AT(160)5 tested at 22 °C and (**f**) AT(160)1 tested at −196 °C.

**Figure 17:**
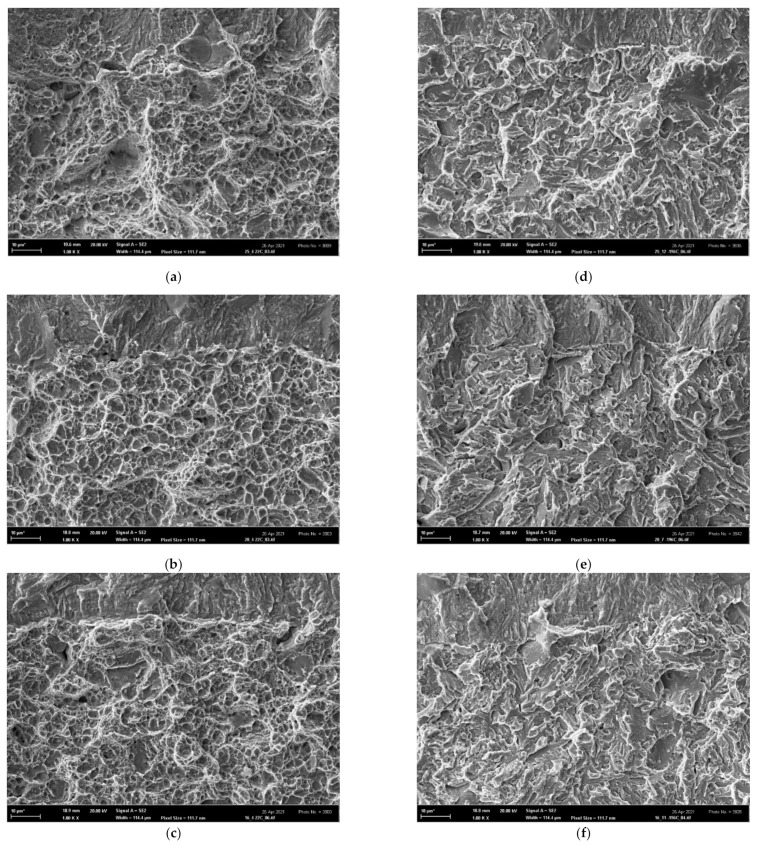

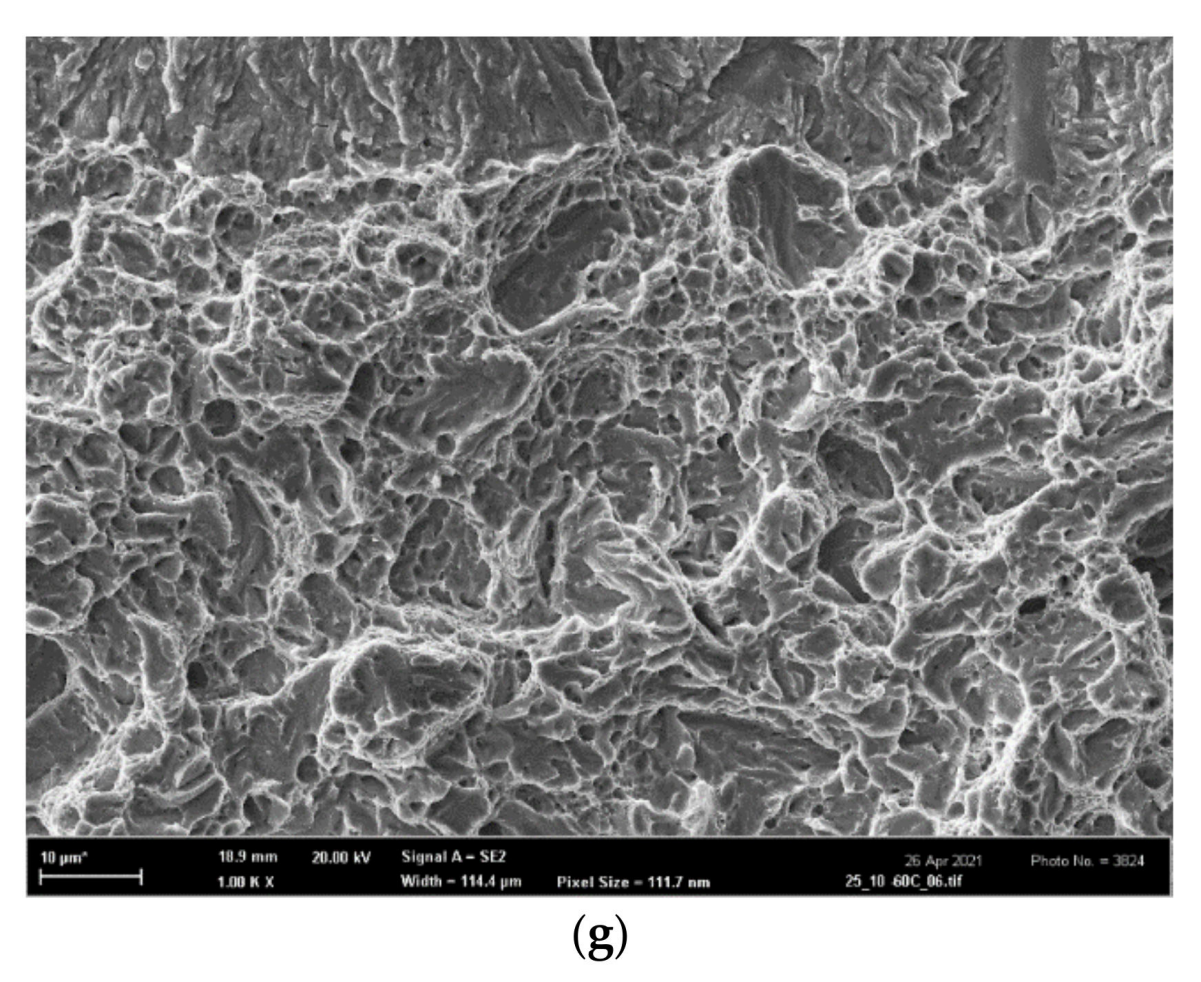
The morphology of FT(X)x samples: the ductile dimple morphology of (**a**) FT(250)5, (**b**) FT(200)5, (**c**) FT(160)5 tested at 22 °C; the ductile-brittle morphology of (**d**) FT(250)1, (**e**) FT(200)1 (**f**) FT(160)1 tested at −196 °C, and (**g**) the ductile-dimple morphology with some cleavage facets of FT(250)3 tested at −60 °C.

On the other hand, all fracture surfaces of the tensile AT specimens were clearly of a ductile dimple morphology in the entire temperature range 〈−196; 22〉 °C, as shown in Figure 16. This documents an extended void-growth inside the well-developed necks, leading to superior tensile characteristics *RA* and *ε*_f_ of all AT specimens. The fracture surfaces of all additionally treated fracture-toughness FT(X) samples in the temperature range 〈−60; 22〉 °C also exhibited a prevalent ductile dimple morphology (similar to the FT(O) samples) as documented in Figure 17a–c for the room temperature tests. At the temperature of −60 °C, however, some cleavage facets could already be observed as documented in Figure 17g for the FT (250) sample. The fracture morphology of FT(X) samples at the lowest temperature was of a mixed ductile/brittle or quasi-brittle character as depicted in Figure 17d–f. The much less ductility observed in the FT(X) samples compared to the AT(X) ones was a consequence of high tensile stress triaxiality at the pre-crack fronts in the FT(X) specimens. It accelerated the austenite/martensite strain-induced phase transformation, which, particularly during the low-temperature FT tests, quickly transferred the two-phase microstructure to a one-phase martensite structure similar to that of the FT(O) specimens. In the FT samples of all grades, moreover, the high stress triaxiality substantially reduced the values of the fracture strain compared to those in the tensile specimens, thus diminishing their influence on the values of the fracture toughness (see the *ε*_f_-values of the OT samples in Table 4 and the *ε*_fc_-values in Table 7. This corresponds to a higher initial. Both these effects led to a similarity in the fracture behavior of the FT(O) and FT(X) samples and caused a substantial difference in the morphology between AT specimens (ductile-dimple) and FT(X) specimens (quasi-brittle) as observed particularly in the low-temperature range. In contrast to the OT and FT(O) samples, therefore, there was a great difference in the fracture surface morphology of the AT and FT(X) specimens, which clearly indicated a dissimilarity of the fracture mechanisms in the tensile and FT tests of the additionally treated grades.

## 6. Theoretical Prediction and Interpretation of Fracture Toughness Values

Practically all the tested tensile and FT samples exhibited either a prevalent ductile-dimple morphology or at least some ductile markings. Therefore, models based on ductile fracture mechanisms were chosen to be more relevant for a prediction of FT and its temperature dependence. Among the ductile approaches mentioned in the introduction, only the models [16,17,18,19,20] provide theoretical formulae useful for prediction of FT in a straightforward manner. The principles of these models are described in Appendix A.1 and Appendix A.2 and their capability to predict experimental data and reflect the fracture mechanisms is presented hereafter.

The first model (Appendix A.1) enables a prediction of the temperature dependence of FT using the following data from tensile tests performed at corresponding temperatures (see Table 4 and Table 5): *A*, *n*, *E*, *ν* = 0.3 (the Poisson ratio), *d_0_*_,_ and *d*. The solution of Equation (A7) yields the *κ*_c_ value to obtain the fracture strain *ε*_fc_ by introducing *κ* = *κ*_c_ into Equation (A5) and, finally, to predict the *K*_Ic_ value from Equation (A3). The values of *κ*_c,_ *ε*_fc_ along with the predicted *K*_Ic_ values (denoted *K*_Ic_^pr^) for the original steel grades are collected in Table 7 and a graphic comparison of the predicted and experimental values is presented in Figure 11. The agreement between the predicted and valid experimental values is acceptable, which implies that the fracture mechanisms in the tensile and FT tests were similar in the whole temperature range. Predicted and experimental data are covered by the 95% confidence band constructed according to the Master curve concept [34] as also shown in Figure 11.

On the other hand, the prediction of the temperature dependence of FT completely failed for the additionally treated grades. The predicted values were more than two times higher than the experimental data and they did not decrease with decreasing temperature. This is caused by the fact that the fracture mechanisms in the tensile and FT tests were different. Indeed, the ductile-dimple fracture mechanism in all tensile tests at all testing temperatures was associated with extremely high ultimate strength and fracture strain. It corresponded to very high values of the plastic work to fracture according to Equation (A2) while the more brittle fracture mechanism in the real FT tests demanded much less fracture energy. Consequently, the FT values predicted from Equation (A3) were within a highly overestimated range of (150; 300) MPa m^1/2^ in the whole temperature range (−196; 22) °C.

**Figure 18:**
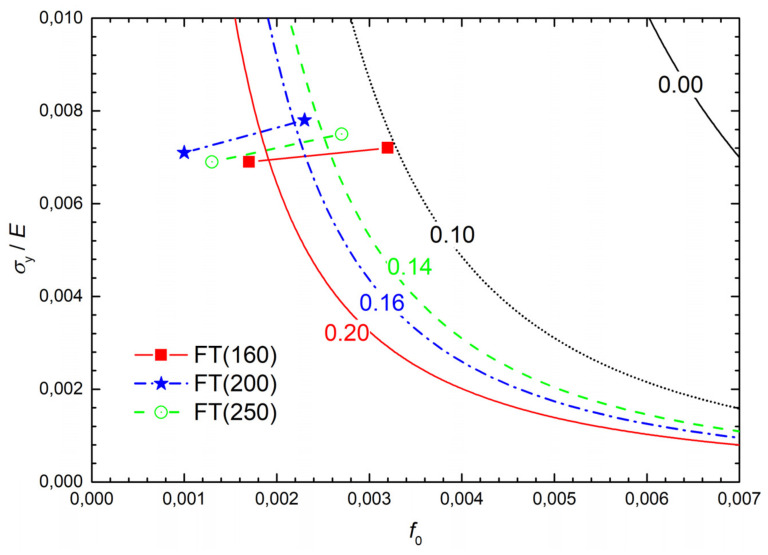
Linear trajectories FT(X) of additionally treated steel grades in the *σ*_y_/*E* vs. *f*_o_ diagram. The corresponding transition curves are labelled by the values of the related hardening exponent *N*.

One can see, however, that the experimental room temperature FT(X) values of the additionally treated grades were nearly two times greater than those pertaining to the lowest temperature. This indicates that the transition from the high-energy ductile fracture to the low-energy tearing with decreasing temperature and increasing yield stress could be assessed in terms of a transition from the single-void model of Rice and Johnson (R-J) [16] to the multiple-void model of Tvergaard and Hutchinson (T-H) [20]; see he Appendix A.2 in more detail. In the diagram *σ*_y_/*E* vs. *f*_o_, where *f*_o_ is the initial area void fraction, such a transfer is represented by a trajectory crossing the transition curve from its left- to the right-hand site. The transition curves in the diagram *σ*_y_/*E* vs. *f*_o_ are related to steel grades by their *N* values. The chart of transition curves for various *N* depicted in Figure 18 was obtained by refining the originally published courser chart in Figure 12 [20] by employing an empirical nonlinear interpolation technique (Figure 12 contains only the transition curves for *N* = 0, 0.1, and 0.2). When a combination of *σ*_y_ and *f*_o_ lies within the single-void regime to the left of the relevant transition curve in Figure 18, then the decreasing temperature (raising *σ*_y_) derives the combination of *σ*_y_ and *f*_o_ across the transition into the low-energy fracture regime. Crossing the transition results in a drop in *K*_Ic_ by a factor of two assuming that the yield stress *σ*_y_ was increased by less than about a factor of 3. Let us now describe the construction of trajectories (straight lines) in the *σ*_y_/*E* vs. *f*_o_ diagram in Figure 18 for additionally treated steel grades to see whether they cross the related transition curves.

**Figure 19:**
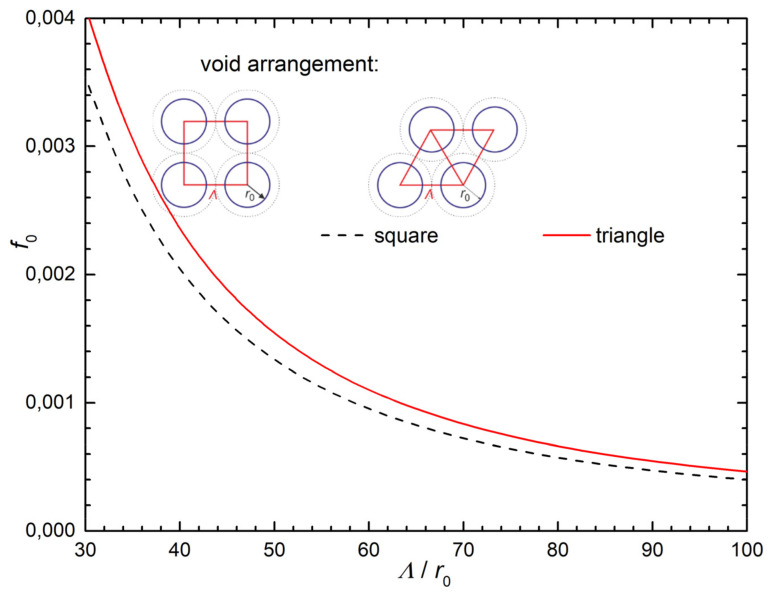
The area void fraction *f*_o_ as a function of the ratio Λ/*r*_o_, where Λ is the mean distance of voids and *r*_o_ is the (initial) void radius for the triangle and square geometrical arrangements of voids.

Given that all steel grades exhibit a single void fracture mechanism at room temperature, the corresponding points in the diagram *σ*_y_/*E* vs. *f*_o_ must lie to the left of each related transition curve, i.e., the void fraction *f*_o_ should be 0.001 in the order of magnitude and c = 2 in Equation (A13). The values of the averaged void spacing Λ for FT(X) samples for 22 °C calculated from Equation (A13) as:

(1) $$\Lambda =\frac{1-\nu^{2}}{2Ec\sigma_{y}}K_{Ic}^{2}$$

are collected in Table 8. Note that these values of 2–3 µm correspond well with the averaged distance between the void centers as documented by the SEM fractography in Figure 17a–c for samples fractured at room temperature. The area void fraction *f*_o_ as a function of the ratio Λ/*r*_o_, where Λ is the mean distance of voids and *r*_o_ is the (initial) void radius, is plotted in Figure 19 for both triangle and square geometrical arrangements of the voids. From this graph, the values of *f*_o_ for all grades can be obtained when considering the Λ values from Table 8 and selecting *r*_o_ = 0.05 µm. These values correspond to the initial (room temperature) points (*f*_o,_ *σ*_y_/*E)* of the trajectories in Figure 18 and ensure that these points lie in the R-J part of the diagram *σ*_y_/*E* vs. *f*_o_, i.e., to the left of the relevant transition curves.

The final points of the trajectories correspond to the testing temperature of −196 °C. Given that they should lie in the T-H part, the averaged void spacing should be determined from Equation (A11) as:

(2) $$\lambda =\frac{1-\nu^{2}}{EW\sigma_{y}}K_{Ic}^{2},$$

where *W* is the work of separation per unit area divided by the product *σ_y_λ* (see Table 8). These values were obtained from Figure 10 in [20] with respect to the *N* values for individual grades. The values of *λ* computed from Equation (2) and shown in Table 8 are slightly lower than those of Λ. This corresponds to a higher initial void fraction *f*_o_ (associated with the higher yield stress *σ*_y_ at −196 °C) and to higher values of *σ*_y_/*E* in Table 8. The final points for −196 °C determine the trajectories of additionally treated grades plotted in Figure 18. Note that the full-line (red) trajectory for the FT(160) grade crosses the associated (red and full-line) transition curve labelled by the value *N* = 0.20, corresponding well with the N values for the AT(160)x samples in Table 5. This also holds for both the FT(200) and FT(250) grades related to the dashed-line (blue) and dashed-and-dot line (black) trajectories and transition curves for *N* = 0.16 and *N* = 0.14, respectively. Thus, all the trajectories for additionally treated grades cross the related transition curves from their left- to the right-hand side, which indicates a change of the fracture mechanisms in terms of the R-J and T-H models. The major part of the trajectory for the FT(160) grade lies in the low-energy fracture region, which corresponds to the occurrence of shallow dimples and cleavage facets on the fracture surfaces in the major part of the temperature range. The opposite is true for both the FT(200) and FT(250) grades, which matches the well-developed dimple fracture morphology observed even at low testing temperatures.

## 7. Conclusions

The results of the experimental investigation of the fracture characteristics of standard heat-treated low-alloyed steel OCHN3MFA along with its three additionally heat-treated grades were presented for the testing temperature range of 〈−196; 22〉 °C. Modelling of the microstructure and fracture processes supported by X-ray and EBSD measurements enabled a physical interpretation of the obtained results, which can be summarized in the following points:

(i) All the additional heat treatments (annealing 650 °C, quenching and tempering to 160, 200, or 250 °C) transferred the standard steel from high- to ultrahigh strength levels even with an improved tensile ductility. The higher strength of the additionally treated grades could be understood in terms of the Hall–Petch relation since they exhibited finer regions of martensitic blocks with a similar crystallographic orientation. The higher ductility could be explained by their two-phase microstructure (martensite + retained austenite) and a more homogeneous distribution of the shape ratio of martensitic laths. A common reason for the increase in both the strength and the ductility was, most probably, the detected reduction of the inclusion content.
(ii) The values of the fracture toughness of all grades were found to be comparable in the whole temperature range due to a high tensile stress triaxiality localized at the process zone ahead of the pre-crack front. It accelerated the strain-induced transformation of austenite to martensite during fracture-toughness tests of the additionally treated grades. Moreover, the triaxiality three-times reduced the fracture strain (compared with the tensile test), thus diminishing its influence on the fracture toughness values.
(iii) The values of the fracture toughness of the standard steel grade could be predicted well using a fracture model proposed by Pokluda et al. based on the tensile characteristics. Such a prediction, however, failed in the case of the additionally heat-treated grades due to their extremely high fracture strains in the tensile tests and different temperature dependence of the fracture mechanisms in the tensile and fracture-toughness tests. While the tensile samples fractured in a ductile-dimple mode at all temperatures, the fracture-toughness specimens exhibited a transition from the ductile to a quasi-brittle fracture mode with a decreasing temperature. This transition was described in terms of a transfer from the void-crack interaction model of Rice and Johnson to the multi-void tearing model of Tvergaard and Hutchinson.

## Figures and Tables

**Table 1:** Chemical composition in wt %.

	C	Mn	Si	Cr	Ni	Mo	V	P	S
Spectral analysis	0.403	0.3	0.32	1.19	3.275	0.523	0.1363	0.01	0.01

**Table 2:** Heat treatment of OCHN3MFA grades: (a) the original grade-conventional treatment; (b) additionally heat-treated grades.

(a)	*Quenching:* heating to 870 ± 10 °C: 2.5 h + 3 h dwell time. Cooling to 300 °C (water) in 3 min. + cooling to room temperature (oil) in 2 h. *Tempering (stepwise):* 480 °C/5 h + 420 °C/5 h.
(b)	*Annealing:* 650 °C/5 h. *Quenching:* 860 °C/1 h., cooling in oil. *Tempering alternatives:* 160 °C/5 h, 200 °C/5 h, or 250 °C/5 h.

**Table 3:** The weighted arithmetic mean *A*_ex_ and the median *M*_ex_ obtained from experimental CDF_ex_ functions and parameters *μ* and *σ* of the related log-normal CDF_LN_ functions.

Sample	OT1	OT2	AT1	AT2
*A*_ex_	0.66358	0.75314	1.07984	1.00900
*M*_ex_	0.25688	0.25688	0.89908	0.77064
*μ*	−1.35914	−1.35914	−0.10638	−0.26053
*σ*	1.37770	1.46673	0.60530	0.73415

**Table 4:** Basic mechanical properties of all steel grades at various testing temperatures.

Sample	*t*_exp_	*E*	*σ*_y_	*σ*_u_	*A*_gt_	*A*_t_	*RA*	*d*_0_	*d*	ε_f_
	°C	GPa	MPa	MPa	%	%	%	mm	mm	%
AT(160)1	−196	236.8	1706	2644	7.3	10.3	31.1	3.914	3.250	37.2
AT(160)2	−120	233.7	1440	2375	6.6	10.3	46.1	3.949	2.900	61.8
AT(160)3	−60	232.5	1422	2287	5.6	8.2	44.3	3.954	2.950	58.6
AT(160)4	−20	228.5	1429	2273	6.0	8.8	40.5	3.914	3.020	51.9
AT(160)5	22	212.4	1468	2250	6.6	9.8	40.9	3.955	3.040	52.6
AT(200)1	−196	239.6	1876	2456	5.9	9.1	37.3	3.929	3.110	46.8
AT(200)2	−120	230.9	1543	2253	6.1	10.1	46.2	3.941	2.890	62.0
AT(200)3	−60	220.6	1479	2127	4.6	6.2	44.0	3.927	2.940	57.9
AT(200)4	−20	211.3	1473	2058	4.1	4.6	45.8	3.938	2.900	61.2
AT(200)5	22	208.6	1487	2111	6.6	10.3	40.3	3.936	3.040	51.7
AT(250)1	−196	241.4	1820	2317	5.7	10.1	36.6	3.944	3.140	45.6
AT(250)2	−20	232.7	1589	2068	4.9	8.7	43.2	3.942	2.970	56.6
AT(250)3	−60	223.7	1529	1998	4.8	8.9	45.7	3.949	2.910	61.1
AT(250)4	−20	222.0	1472	1945	4.4	9.0	50.4	3.919	2.760	70.1
AT(250)5	22	211.3	1448	1902	4.8	9.4	46.8	3.950	2.880	63.2
OT01	−196	244.0	1833	2023	8.0	8.7	12.9	3.986	3.720	13.8
OT03	−100	238.2	1324	1580	5.7	6.3	31.9	4.011	3.309	38.5
OT04	−80	232.1	1384	1631	5.4	10.8	34.2	3.984	3.232	41.8
OT05	−60	226.6	1380	1609	5.6	10.8	32.0	3.995	3.295	38.5
OT12	22	209.4	1309	1523	4.7	10.1	36.9	3.993	3.173	46.0

**Table 5:** Parameters *A* and *n* of the Hollomon approximation and the parameter *N* of the T-H function for all steel grades and testing temperatures.

Sample	*A*	*n*	*N*
	MPa	-	-
AT(160)1	3733.3	0.124	0.19
AT(160)2	3495.7	0.135	0.21
AT(160)3	3417.8	0.131	0.21
AT(160)4	3472.9	0.137	0.21
AT(160)5	3621.6	0.150	0.20
AT(200)1	3560.6	0.114	0.14
AT(200)2	3145.0	0.115	0.16
AT(200)3	3022.8	0.111	0.17
AT(200)4	2931.7	0.105	0.17
AT(200)5	2811.7	0.099	0.15
AT(250)1	2939.1	0.077	0.12
AT(250)2	2728.9	0.085	0.13
AT(250)3	2625.7	0.083	0.13
AT(250)4	2607.6	0.086	0.14
AT(250)5	2532.8	0.085	0.14
OT01	2284.5	0.027	0.09
OT03	1492.7	0.040	0.05
OT04	1829.1	0.038	0.08
OT05	1839.9	0.042	0.07
OT12	1951.7	0.064	0.09

**Table 6:** Experimental values of the fracture toughness at all testing temperatures.

	Original Steel Grade				Additionally Treated 160 °C		Additionally Treated 200 °C		Additionally Treated 250 °C	
*t*_exp_	Sample	*K*_exp_	Sample	*K*_exp_	Sample	*K*_exp_	Sample	*K*_exp_	Sample	*K*_exp_
°C		MPa m^1/2^		MPa m^1/2^		MPa m^1/2^		MPa m^1/2^		MPa m^1/2^
−196	FT(O)1	23.3	FT(O)2	22.9	FT(160)1	24.2	FT(200)1	27.5	FT(250)1	24.5
−120					FT(160)2	34.3	FT(200)2	38.7	FT(250)2	33.0
−100	FT(O)3	46.6								
−80	FT(O)4	49.2								
−60	FT(O)5	56.4	FT(O)6	61.4	FT(160)3	38.2	FT(200)3	47.0	FT(250)3	45.8
−50	FT(O)7	57.8								
−40	FT(O)8	61.4	FT(O)9	72.5 †						
−20	FT(O)10	72.0 *	FT(O)11	73.1 *	FT(160)4	44.8	FT(200)4	52.4	FT(250)4	52.5
22	FT(O)12	69.1 *			FT(160)5	47.8	FT(200)5	56.2	FT(250)5	60.5

Note: * K_JU_; † K_JC_.

**Table 7:** Values of *κ*_c_, *ε*_fc_, and predicted *K*_Ic_^pr^ for original steel grades.

Sample	*κ*_c_	*ε*_fc_	*K*_Ic_^pr^
	-	%	MPa m^1/2^
FT(O)1	1.867	3.4	26.3
FT(O)3	1.521	9.3	50.5
FT(O)4	1.490	10.2	55.1
FT(O)5	1.521	9.4	56.8
FT(O)12	1.454	11.4	92.3

**Table 8:** Parameters Λ, *λ*, *W*, *f*_o_, and *σ*_y_/*E* calculated for the initial and final points of the trajectories related to the additionally treated samples in Figure 18.

Sample	*t*_exp_	Λ	*λ*	*W*	*f*_o_ (Λ)	*f*_o_ (*λ*)	*σ*_y_/*E*
	°C	μm	μm	-	‰	‰	-
FT(160)1	−196		1.63	0.81		3.2	0.0072
FT(160)5	22	2.23			1.7		0.0069
FT(200)1	−196		1.91	0.80		2.3	0.0078
FT(200)5	22	2.90			1.0		0.0071
FT(250)1	−196		1.78	0.70		2.7	0.0075
FT(250)5	22	2.59			1.3		0.0069

## Data Availability

Data supporting the findings of this study are available from the corresponding author upon request.

## Appendix A. Models of Fracture Toughness Based on the Localized Ductile Damage

### Appendix A.1. The Fracture Energy Specified by the Critical Strain in the Process Zone

The model introduced by Staněk and Pokluda [17] and further modified by Pokluda and Šandera [18,19] determines the critical fracture strain in the crack-tip process zone using the so-called *failure locus* as the dependence of the fracture strain on the stress triaxiality for a given material. The related FT value can then be predicted using the hypothesis of linear damage accumulation. This method was developed particularly for the assessment of *K*_Ic_ values from the mechanical characteristics available from tensile tests of smooth and circumferentially notched cylindrical specimens. The method assumes that the crack tip blunting takes place during the initial phase of FT tests and the related damage process localized in the crack-tip plastic (process) zone precedes the unstable fracture. When performing the standard FT test of relatively small deformation rates under conditions of small-scale yielding and plane strain, the dissipation energy in the form of elastic waves can be neglected. Thus, the Griffith–Irwin–Orowan fracture criterion can be considered as:

(A1) $$\frac{1-\nu^{2}}{E}K_{Ic}^{2}=2\gamma +\omega_{p}\left(\varepsilon_{fc}\right),$$

where *γ* is the surface energy, *ω*_p_ (*K*, *γ*) is the fracture energy, i.e., the work per unit area needed for building the plastic zone up to the moment of fracture, and *ε*_fc_ is the critical (local) fracture strain in the process zone. Then, the following physically relevant presumptions are considered [19]:

(i) Nearly all energy supplied by external forces and/or released by elastic relaxation is consumed in the plastic zone during the ductile crack tip blunting process preceding the unstable crack advance. In other words, the second term in Equation (1) is much higher than the first one (*ω*_p_ ≫ *2γ*).
(ii) The unstable fracture is controlled by reaching a critical value of the plastic strain *ε*_fc_ in the process zone at the crack tip.

Given that the Hollomon approximation *σ* = *Aε*_p_^n^ describes the stress-plastic strain behavior during formation of the plastic zone sized *r*_p_ = *Bn*^2^ (*B* ≈ 0.025 m) [12]. Then, the plastic work consumed till the onset of unstable fracture can be expressed as:

(A2) $$\omega_{p}\left(\varepsilon_{fc}\right)=2ABn^{2}\overset{\varepsilon_{fc}}{\underset{0}{\int }}\varepsilon_{p}^{n}\left(\kappa \right)d\varepsilon_{p}$$

and Equation (A1) can be rearranged to:

(A3) $$K_{Ic}={\left[\frac{2ABE\varepsilon_{fc}^{n+1}}{\left(1-\nu^{2}\right)\left(n+1\right)}\right]}^{1/2}.$$

Owing to the highly triaxial tensile stress inside the crack-tip process zone and the void-coalescence fracture mechanism, the value of *ε*_fc_ is much less than the fracture strain *ε*_f_ of smooth tensile samples (e.g., [17,19]) and it must be determined by the following procedure. The dependence of the fracture strain on the stress triaxiality factor *κ* (the ratio of the hydrostatic stress and the von Mises effective stress) can be expressed as:

(A4) $$\varepsilon_{f}\left(\kappa \right)=\frac{4}{5\kappa }\ln\frac{d_{o}}{d}.$$

Equation (A4) is the simplest phenomenological approximation of the fracture locus. During the blunting process, the process zone elements experience a strain trajectory:

(A5) $$\varepsilon_{p}\left(\kappa \right)=7.7\exp\left(-2.9\kappa \right).$$

starting at *κ* = $\left(n+1\right)/\sqrt{3}$ [17,37]. When adopting the hypothesis of the linear damage accumulation, the critical factor *κ*_c_ associated with the onset of unstable fracture is determined by the integral equation:

(A6) $$\overset{\varepsilon_{p}(\kappa_{c})}{\underset{\varepsilon_{p}\left(\left(n+1\right)/\sqrt{3}\right)}{\int }}\frac{d\varepsilon_{p}\left(\kappa \right)}{\varepsilon_{f}\left(\kappa \right)}=\overset{\kappa_{c}}{\underset{\left(n+1\right)/\surd 3}{\int }}-\frac{27.9\;\kappa \;\exp\left(-2.9\kappa \right)}{\ln\left(d_{o}/d\right)}\;d\kappa =1.$$

When combining Equations (A4), (A6), and (A7), one obtains:

(A7) $$\left(7.7\kappa_{c}+2.655\right)\;\exp\left\{-2.9\kappa_{c}\right\}=0.8\;\ln\left(\frac{d_{o}}{d}\right)+0.0205.$$

The iterative solution of Equation (A7) yields the *κ*_c_ value, and the fracture strain *ε*_fc_ is then obtained from Equation (A5) by introducing *κ* = *κ*_c_. Finally, the *K*_Ic_ value is predicted from Equation (A3).

### Appendix A.2. The Separation Energy Related to Rupture of Void Ligaments

The Tvergaard–Hutchinson (T-H) model [20] assumes a pre-existing population of roughly similar sized voids that give rise to an unstable fracture localized to a “void sheet” in the process zone. The void spacing is comparable to the initial thickness λ of the void sheet, the length *l* of which is much larger. The model constitutes a transition from the classical high-energy ductile fracture by a spatial coalescence of voids to the planar coalescence representing a sort of low-energy ductile tearing caused by many small voids interacting on a plane ahead of the crack tip.

The Gurson model [15] for an elastic-plastic solid containing voids is used to predict the traction–displacement law associated with the fracture process zone. The fracture localization occurs in a layer of initial thickness *λ* identified with the average spacing of voids with the area fraction *f*_o_. The yield condition of this model is:

(A8) $$\Phi \left(\sigma_{c},\sigma_{m},f\right)={\left(\frac{\sigma_{c}}{\sigma_{\mathrm{eff}}}\right)}^{2}+2q_{1}f\cosh\left(\frac{3\sigma_{m}}{2\sigma_{\mathrm{eff}}}\right)-[1+{\left(q_{1}f^{2}\right)}^{2}]=0,$$

where $\sigma_{c}$ is the effective stress, $\sigma_{c}$= $\sigma_{kk}/3$ is the mean stress, *σ*_eff_ is the current effective stress associated with the matrix, *f* is the current void volume fraction, and *q*_1_ = 3/2 is the adjustment factor. The traction–displacement relation is computed assuming the failing layer undergoes uniaxial straining in the direction normal to the crack plane. The elastic-plastic solid has a tensile yield stress, $\sigma_{y}$, and a true stress logarithmic strain curve in uniaxial tension is specified by:

(A9) $$\begin{array}{cc} \varepsilon =\frac{\sigma }{E} & \sigma \leq \sigma_{y},\\ \varepsilon =\frac{\sigma_{y}}{E}{\left(\frac{\sigma }{\sigma_{y}}\right)}^{\frac{1}{N}} & \sigma \geq \sigma_{y} \end{array}$$

The work of separation per unit area is then computed as:

(A10) $$\Gamma_{o}=\int_{0}^{\delta_{c}}\sigma \left(\delta \right)d\delta$$

where *σ*(*δ*) is the traction–displacement law. The calculations revealed that the term *W* = Γ_o_/(*σ*_y_ *λ*) only slightly depends on *N* but is insensitive to the area fraction *f*_o_ and its value lies in the range of (0.7, 1). Assuming Γ_o_ ≡ *ω*_p_ in Equation (A1), the T-H model leads to the FT prediction:

(A11) $$K_{Ic}={\left[\frac{W}{\left(1-\nu^{2}\right)}\right]}^{1/2}{\left(E\sigma_{y}\lambda \right)}^{1/2}.$$

This formula is similar to that resulting from the model of Rice and Johnson (R-J) [16], which assumes the ductile fracture mechanism involving an interaction of just one void with the crack tip. The multiple-void T-H model transfers into the single-void R-J model when the geometrical condition *λ*/*l* = 0.5 is fulfilled. In the R-J model, the initiation of crack growth takes place when the void nearest to the tip begins to coalesce with the tip. This will happen when *δ*_t_ = *c*Λ, where *δ*_t_ is the crack tip opening displacement, Λ is the average void spacing, and *c* is a number, which depends weakly on *f*_o_ varying from about 2 for *f*_o_ = 0.001 to about 1 at *f*_o_ = 0.05. Using the small-scale yielding estimate:

(A12) $$\delta_{t}=0.5\left(1-v^{2}\right)K^{2}/{\left(E\sigma_{y}\Lambda \right)}^{1/2},$$

one obtains the R-J prediction of FT as:

(A13) $$K_{Ic}={\left[\frac{2c}{\left(1-\nu^{2}\right)}\right]}^{1/2}{\left(E\sigma_{y}\Lambda \right)}^{1/2}.$$

If *λ* in the T-H model is identified with Λ in the R-J model, then (A11) and (A13) differ only in their numerical coefficients. The coefficient in *K*_Ic_ for the R-J model is in the range of about two times that for the T-H model.

Note that both the T-H and R-J models predict an increase in *K*_Ic_ with increasing *σ*_y_ when lowering the testing temperature, but the opposite effect is commonly observed. Even if the initial void volume fraction *f*_o_ is supposed to increase with $\sigma_{y}$ due to greater void nucleation at the higher stress, the dependence of each prediction on *f*_o_ is too weak to result in a drop in *K*_Ic_. A possible explanation for such a drop may be a transition from the single- to the multiple-void mechanism associated with the condition *λ*/*l* = 0.5. This condition corresponds to transition curves for different values of *N* in the diagram *σ*_y_/*E* vs. *f*_o_ [20]. The transfer from the single- to multiple-void mechanism then corresponds to crossing these curves from the left- to the right-hand site; see the Section 6 of this article for more detail.
